# Transnational prenatal care among migrant women from low-and-middle-income countries who gave birth in Montreal, Canada

**DOI:** 10.1186/s12884-023-05582-w

**Published:** 2023-04-26

**Authors:** Lisa Merry, Ye Na Kim, Marcelo L. Urquia, Julie Goulet, Sarah Fredsted Villadsen, Anita Gagnon

**Affiliations:** 1 Faculty of Nursing, University of Montreal, Montreal, Canada; 2 Manitoba Centre for Health Policy, Rady Faculty of Health Sciences, University of Manitoba, Winnipeg, Manitoba, Canada; 3 School of Psychoeducation, University of Montreal, Montreal, Canada; 4 Department of Public Health, University of Copenhagen, Copenhagen, Denmark; 5 Ingram School of Nursing, Faculty of Medicine, McGill University, Montreal, Canada

**Keywords:** Maternity care, Migrants, Migrant health, Prenatal care, Reproductive health, Transnationalism

## Abstract

**Objectives:**

There is little research examining transnational prenatal care (TPC) (i.e., prenatal care in more than one country) among migrant women. Using data from the *Migrant-Friendly Maternity Care (MFMC) - Montreal project*, we aimed to: (1) Estimate the prevalence of TPC, including TPC-arrived during pregnancy and TPC-arrived pre-pregnancy, among recently-arrived migrant women from low- and middle-income countries (LMICs) who gave birth in Montreal, Canada; (2) Describe and compare the socio-demographic, migration and health profiles and perceptions of care during pregnancy in Canada between these two groups and migrant women who received no TPC (i.e., only received prenatal care in Canada); and (3) Identify predictors of TPC-arrived pre-pregnancy vs. No-TPC.

**Methods:**

The MFMC study used a cross-sectional design. Data were gathered from recently-arrived (< 8 years) migrant women from LMICs via medical record review and interview-administration of the MFMC questionnaire postpartum during the period of March 2014-January 2015 in three hospitals, and February-June 2015 in one hospital. We conducted a secondary analysis (n = 2595 women); descriptive analyses (objectives 1 & 2) and multivariable logistic regression (objective 3).

**Results:**

Ten percent of women received TPC; 6% arrived during pregnancy and 4% were in Canada pre-pregnancy. The women who received TPC and arrived during pregnancy were disadvantaged compared to women in the other two groups (TPC-arrived pre-pregnancy and No-TPC women), in terms of income level, migration status, French and English language abilities, access barriers to care and healthcare coverage. However, they also had a higher proportion of economic migrants and they were generally healthier compared to No-TPC women. Predictors of TPC-arrived pre-pregnancy included: ‘Not living with the father of the baby’ (AOR = 4.8, 95%CI 2.4, 9.8), ‘having negative perceptions of pregnancy care in Canada (general experiences)’ (AOR = 1.2, 95%CI 1.1, 1.3) and younger maternal age (AOR = 1.1, 95%CI 1.0, 1.1).

**Conclusion:**

Women with more capacity may self-select to migrate during pregnancy which results in TPC; these women, however, are disadvantaged upon arrival, and may need additional care. Already-migrated women may use TPC due to a need for family and social support and/or because they prefer the healthcare in their home country.

**Supplementary Information:**

The online version contains supplementary material available at 10.1186/s12884-023-05582-w.

## Background

Large numbers of women travel across international borders during their reproductive years and experience pregnancy and childbirth in high-income countries [1, 2]. Although prenatal care is important in supporting a healthy pregnancy and reducing adverse outcomes, studies reveal that many migrant women (e.g., immigrants, refugees) either do not adequately utilize prenatal health services or receive poorer quality care in the destination country [3, 4]. From 2011 to 2016 Canada received over one million permanently settling migrants, and it is expected that by 2036 migrants will make up 25 to 30% of the population [5]. The vast majority of migrants are arriving from low- and middle-income countries (LMICs) and resettling in major urban centers [5]. In Montreal, one of the main receiving cities, migrants represent more than half of the women giving birth, stressing the need to understand prenatal care use and experiences among these women [6].

Migrant women often face significant barriers to maternity care including delayed access to, or exclusion from public health insurance, and marginalization due to their migration status [3, 7, 8]. Resettlement challenges (e.g. finding employment) and the loss of traditional support systems can also cause delays in seeking care [9, 10]. Within healthcare encounters, migrant women may face language and communication barriers, cultural insensitivity, and discrimination due to their religious affiliation or membership in a racialized group [8, 9, 11–13]. Many childbearing migrant women report receiving insufficient information during pregnancy or at the time of birth or have difficulties understanding the information received, and also contend with challenges navigating the healthcare system [8, 10, 11, 14, 15]. Migrant women also express feeling unwelcomed, rushed, and a lack of kindness on the part of healthcare providers [7, 10, 11, 14].

Given the number of barriers that migrant women confront while accessing maternity care in destination countries, it is expected that they may engage in and rely on transnational healthcare to maintain their health and well-being during pregnancy. Transnational healthcare behaviors include purchasing medications and receiving health advice and information through virtual networks and/or traveling to another country to directly obtain healthcare and support; the latter is affected by migration status and the other types of transnational ties [16]. Having a precarious legal status (e.g. undocumented migrants) reduces the likelihood of seeking transnational health services due to the risk and fear of not being granted re-entry into the destination country after leaving [17]. Whereas migrants who maintain social ties with family and friends in their country of origin, and/or who are less integrated into the host country (i.e., have fewer social connections, are less fluent in the host country’s language and have been in the country for a relatively short period of time), are more likely to travel and seek healthcare and support abroad [18, 19]. Research has shown that migrants may access healthcare in other countries to overcome barriers in the receiving country [16–24]. Migrants have stated that their negative perceptions of the destination country’s healthcare system, and the affordability of, and personal comfort in the health services of the country of origin, encourage them to seek out transnational healthcare [19, 22–24].

To our knowledge, no quantitative studies have examined transnational pregnancy healthcare use, including the prevalence and factors associated with migrant women seeking prenatal care abroad. However, qualitative studies with Pakistani and Bengali migrant women in the United States found that some women make return visits to their origin country during their pregnancy to spend time with family and to receive ‘special care’, while others receive guidance and help via visits from their mothers [25, 26]. Migrant women also reported receiving gifts and goods sent from abroad and using phone calls to obtain informational and emotional support from family members. Using social media and technology to obtain pregnancy advice and health information virtually was also shown in another qualitative study with Caribbean migrant women living in the United States [27]. A recurring theme across this research is women feeling that they lack family connection and support in the receiving-country and that the pregnancy care is insensitive to their social and cultural context; these women therefore long for the care and support back home [25–28].

Transnational healthcare use during pregnancy may also be the result of women migrating during pregnancy. The profiles, and experiences and perceptions of healthcare in the destination country among these women, may differ when compared to migrant women who have actively sought care transnationally, or who have not received transnational care at all. We found no studies that focused specifically on women who experienced transnational prenatal care associated with migration during pregnancy. In general, research suggests that it may be women in more vulnerable contexts (e.g., asylum-seekers) who are more likely to migrate during pregnancy [29, 30]. These women however are also more likely compared to other migrants to face challenges accessing healthcare pre- and during migration and to arrive in the destination country without having received any pregnancy care at all [29, 30]. Also, compared to women who have been in the country for a longer period of time, women who arrive during pregnancy seem to be more likely to experience delays or inadequate prenatal care in the new country [14, 30], likely due to unfamiliarity with the healthcare system or resettlement barriers. Despite having interrupted or inadequate prenatal care, these women however, may be healthier because of the ‘healthy immigrant effect’ (i.e., the phenomenon where migrants are healthier than the receiving-country population upon arrival) [31–33]. Regarding perceptions of care, they may have more positive or neutral views given the little interaction they have had with the healthcare system and/or from drawing comparisons between their health experiences in the new country with that of their country of origin, which may have been a resource-poor setting [23, 34]. Migrant women with more precarious statuses may also not express dissatisfaction or negative views due to fear of it affecting their immigration process.

Migrant women’s transnational healthcare use during pregnancy may affect their health and shape their health-related behaviors, including healthcare-seeking in the destination country [16], which can have implications on pregnancy, birth and infant outcomes. The rapid advancements in information and communication technology have facilitated the exchange of information across borders. The movement of goods and people between countries is also easier than it has ever been. In the context of our increasingly globalized world, there is a need to study and gain a better understanding of transnational healthcare use among pregnant migrant women.

The Migrant-Friendly Maternity Care (MFMC) – Montreal study is a study that aimed to examine perceptions of maternity care among recently-arrived migrant women from LMICs giving birth in Montreal, Canada during the period of 2014–2015 [35]. Although detailed information was not gathered on the various types of transnational healthcare use, women in this study were asked in which countries they had received prenatal care by a healthcare professional. This dataset therefore makes it possible to begin to investigate transnational healthcare use among pregnant migrant women. Using the MFMC study data, the objectives of the present study were to:

1. Estimate the prevalence of transnational prenatal care (TPC), including TPC-arrived during pregnancy and TPC-arrived pre-pregnancy;
2. Describe and compare the socio-demographic, migration and health profiles and perceptions of care during pregnancy in Canada between TPC-arrived during pregnancy, TPC-arrived pre-pregnancy and No-TPC (i.e., migrant women who only received prenatal care in Canada) women; and
3. Identify predictors of TPC-arrived pre-pregnancy vs. No-TPC.

## Methods

**Fig. 1:**
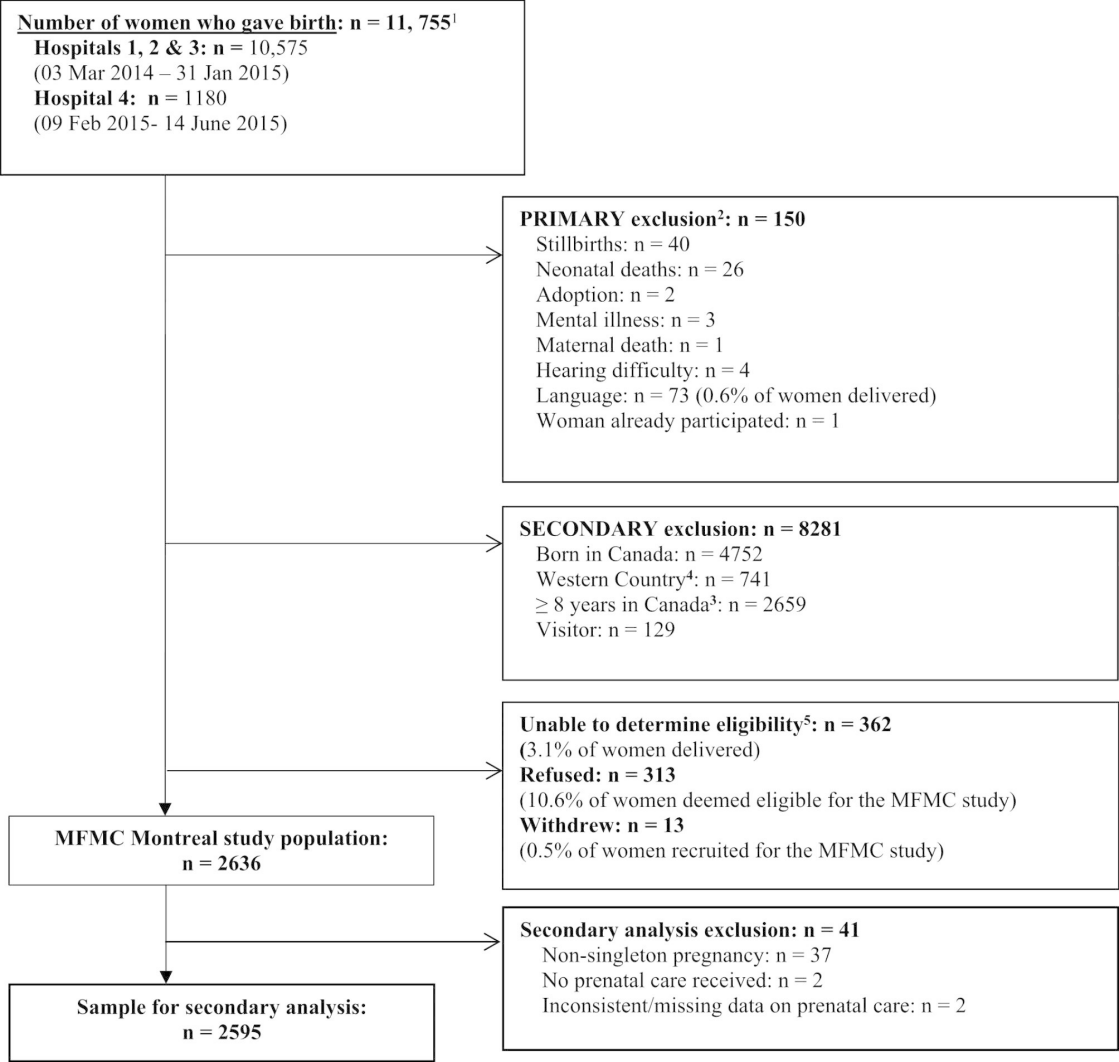
Study sample **Legend**: Note: MFMC = Migrant-Friendly Maternity Care ^1^ 227 twins and 7 sets of triplets ^2^ Primary MFMC exclusion criteria determined by hospital birthing log and/or medical chart or if unable to communicate with woman (i.e., exclusion did not require administration of a questionnaire) ^3^ Initially exclusion was > 5 years. It was changed to ≥ 8 years after 5 months of recruitment to optimize the recruitment rate ^4^ From the United States, Northern or Western Europe, Scandinavia, Israel, Australia, New Zealand or Japan ^5^ Women were discharged or declined to be approached or research personnel were restricted by hospital staff to approach women

We conducted a secondary analysis using data from the MFMC study. The MFMC study used a cross-sectional design. Data were gathered from 2636 recently-arrived (defined as < 8 years in Canada) migrant women from LMICs (i.e. not from the United States, Northern or Western Europe, Oceania, or Japan), who gave birth in one of four hospitals, which had the greatest number of birthing migrant women in Montreal (i.e., at the time of the study 55–71% of women giving birth at these hospitals were migrants). All women who gave birth during the recruitment period (March 2014 to January 2015 for three hospitals, and February 2015 to June 2015 for one hospital), were screened for eligibility. Details on the number of women considered for participation and those excluded, are provided in Fig. 1.

Data in the MFMC study were collected from medical records and through use of an interview-administrated questionnaire while women were still in hospital postpartum. The questionnaire used was the Migrant-Friendly Maternity Care Questionnaire (MFMCQ) [36], which was validated and translated into eight languages (Arabic, Punjabi, Vietnamese, Chinese (Mandarin), Urdu, Hindi, Spanish, and French); the English version can be viewed through the following link: https://static-content.springer.com/esm/art%3A10.1186%2F1471-2393-14-200/MediaObjects/12884_2013_1090_MOESM2_ESM.pdf ; additional questions were added for the MFMC study, these are available in Additional File 1. The questionnaire was used to obtain socio-demographic (e.g., income, education, marital status), migration (e.g., country of birth, migration status, languages spoken) and general health characteristics, and women’s perceptions on care during pregnancy, childbirth and early postpartum. Data collected from medical records included obstetrical and maternal and infant health information. Missing data were minimal, less than 3% for all variables, except for the income variable; 16% of women did not provide a response for this question.

For the secondary analysis, we restricted the sample to women who had a singleton pregnancy and reported receiving prenatal care from a healthcare professional. Two women were subsequently excluded due to inconsistencies and missing data on prenatal care. The final sample for the secondary analysis was 2595 women.

For objective one (estimation of prevalence of TPC), we divided the sample into three groups based on whether or not they had received TPC and the timing of their migration and arrival in Canada (pre-pregnancy vs. during pregnancy). TPC was determined based on the MFMCQ question four: “*Did you receive care for this pregnancy from a healthcare professional (such as a doctor, nurse, or midwife)? And if yes, in which countries*?” Women who responded yes, and who indicated Canada, and at least one other country as locations of care, were categorized as having had TPC; two women, however, were also considered as ‘transnational’ although they had only received prenatal care outside of Canada due to migration in very late pregnancy. We then further divided this group into two based on the assumption that women who arrived during pregnancy had received TPC as a result of their migration during pregnancy, while women who were in Canada pre-pregnancy had received TPC because they had intentionally sought out care abroad. Length of time in Canada (question two on the MFMCQ) was used to determine whether women had migrated during pregnancy; length of time ≤ 10 months was considered ‘migrated and arrived during pregnancy’ and length of time > 10 months was considered ‘in Canada pre-pregnancy’. Question three on the MFMCQ (“*Did you arrive in this country pregnant with the recent baby?*”) was used to verify this categorization. There were 18 women who reported receiving TPC and arriving during pregnancy, but whose length of time in Canada was > 10 months at the time of giving birth. The length of time variable was deemed to be more reliable since it was collected twice, once to determine eligibility for participation in the MFMC study and then again during data collection. We were also able to compare the length of time in Canada data to another question which asked about age of arrival in Canada. After verification, these 18 women remained classified as TPC-arrived pre-pregnancy.

To address objective two (comparison of profiles and perceptions of care), we performed descriptive analyses by TPC group (TPC-arrived during pregnancy; TPC-arrived pre-pregnancy; and No-TPC women). Analyses included socio-demographic, migration, and health characteristics and perceptions on care received during pregnancy in Canada. We also tested for differences between groups using Pearson chi-square test for categorical data and ANOVAs for continuous variables; post hoc analyses were done to identify the differences between the three groups using least significant difference for ANOVAs and column proportion test using Z-test for Pearson chi-square tests.

For objective three (predictors of TPC-arrived pre-pregnancy), we used logistic regression modelling to identify socio-demographic, migration, health, and perceptions of care variables significantly associated to TPC-arrived pre-pregnancy; No-TPC women served as the reference group. Unadjusted and adjusted odds ratios (AOR) using 95% confidence intervals (CI) were estimated. The multivariable model was constructed using the ‘purposeful selection of covariates’ approach as described by Hosmer, Lemeshow, and Sturdivant, (2013) [37]. Variables were selected based on the literature and theoretical relevance. Variables initially included in the model were: parity, maternal age, length of time in Canada, region of origin, living with the father of the baby, maternal education level, paid for medical services during pregnancy, pregnancy complications, perceptions of pregnancy care in Canada (general experiences), and perceptions of pregnancy care in Canada (language/communication related); the two perceptions’ variables were composite variables (Cronbach’s alpha for general experiences, α = .696, for language/communication, α = .572). The details on how each variable was operationalized are available in Additional File 2. We also constructed a model excluding the 18 women who had reported receiving TPC and arriving during pregnancy, but whose length of time in Canada was > 10 months, to see if this would alter the results. Before estimating the multivariable models we assessed for multicollinearity between variables using variance inflation factors and tolerance showing to ensure correlations between the independent variables were not too high [38]. The fit of the model was assessed using the Hosmer-Lemeshow test and the receiver operating characteristic (ROC) curve was used to assess the discrimination ability of the fitted model. All analyses were conducted using SPSS.

## Results

A total of 248 women (9.6%) reported receiving TPC from a healthcare professional; 148 (5.7%) had arrived during pregnancy and 100 (3.9%) were in Canada pre-pregnancy. Two thousand three hundred and forty-seven women (90.4%) received prenatal care from a healthcare professional only in Canada; 2.6% (n = 60) of these women stated they had arrived during pregnancy. Overall (N = 2595), the average age of women was 32 years old (SD = 4.7 years), their education level was high, 85% had a post-secondary education degree, and almost all (97%) were married. Forty-three percent of women were from the Middle-East/North Africa, 16% were from Sub-Saharan Africa, 14% from East/South East Asia, 11% from South America, and the remainder originated from South Asia (8%) and Eastern Europe (7%). The average length of time in Canada was three years (SD = 23.4 months) with a range of one month to 8 years. Forty-six percent of women had come as an economic immigrant, temporary worker or student, 50% were sponsored by a family member, and 4% had a refugee history or were an asylum-seeker or had no status. Sixty-nine percent were fluent in either English and/or French (both languages are spoken in Montreal; French however, is the official language in the province of Quebec).

**Fig. 2:**
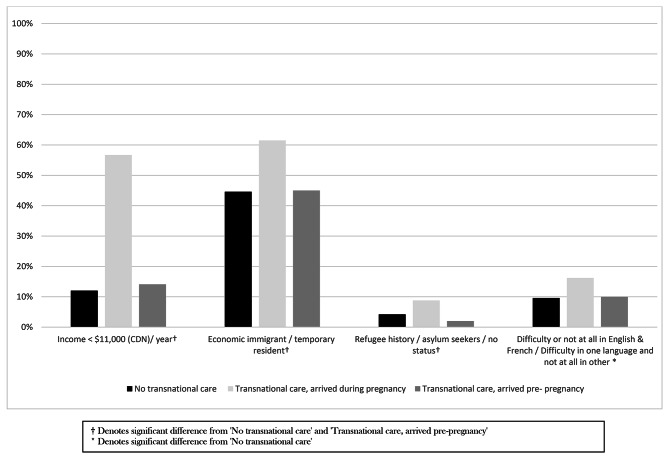
Sociodemographic profiles, Transnational care, arrived during pregnancy women vs. No transnational care and Transnational care, arrived pre- pregnancy women

Socio-demographic and migration characteristics by group are presented in Table 1. There was a statistically greater proportion of women with a very low income (< $11,000 CDN/year) among the TPC-arrived during pregnancy women (57%) compared to the No-TPC (12%) and the TPC-arrived pre-pregnancy (14%) women. Migration status and French/English language ability also differed, with a higher percentage of humanitarian/precarious status migrants (9%) and women with difficulties/no ability in both languages (16%) among the TPC-arrived during pregnancy group compared to the other two groups (4% and 2% of women had a humanitarian/precarious status in the No-TPC and TPC-arrived pre-pregnancy groups respectively; 10% of women in both groups had difficulties/no ability in both languages). Conversely, the TPC-arrived during pregnancy group also had a higher proportion of economic/temporary migrants (62% vs. 45% in each of the other two groups) and a smaller proportion of family sponsored migrants (30% vs. 51% and 53%). The mean length of time in Canada was statistically shorter as well (4 months vs. 41 and 39 months). A summary of sociodemographic differences between TPC-arrived during pregnancy women and No-TPC and TPC-arrived pre-pregnancy women is presented in Fig. 2.

**Table 1:** Socio-demographic and migration characteristics, N=2595

Characteristic	No transnational care *n* = 2347 (90.4%)	Transnational care, arrived during pregnancy *n* = 148 (5.7%)	Transnational care, arrived pre- pregnancy *n* = 100 (3.9%)	*P* value
Age, n (%)				
≤ 25 years	236 (10.1)	12 (8.1)	18 (18.0)^*^	0.024
26–34 years	1457 (62.1)	104 (70.3)	60 (60.0)	
≥ 35 years	654 (27.9)	32 (21.6)	22 (22.0)	
Age, mean years (SD)	32.1 (4.7)	31.7 (4.4)	31.0 (5.0)^*^	0.035
Education, n (%)	*n* = 2346	*n* = 147		
Primary/secondary school	338 (14.4)	13 (8.8)	11 (11.0)	0.177
Postsecondary/graduate diploma	1991 (84.9)	134 (91.2)	89 (89.0)	
None	17 (0.7)	0 (0.0)	0 (0.0)	
Income (CDN $), n (%)	*n* = 2000	*n* = 104	*n* = 85	
< $11,000/ year	241 (12.0)	59 (56.7)^†^	12 (14.1)	
$11,000-$20,999/ year	344 (17.2)	17 (16.3)	14 (16.5)	
$21,000-$40,999/ year	672 (33.6)	11 (10.6)^†^	31 (36.5)	0.000
$41,000-$60,999/ year	384 (19.2)	5 (4.8)^†^	15 (17.6)	
$61,000-$80,999/ year	191 (9.6)	6 (5.8)	6 (7.1)	
≥ $81,000/ year	168 (8.4)	6 (5.8)	7 (8.2)	
Marital status, n (%)	*n* = 2345			
Married/union	2281 (97.3)	145 (98.0)	97 (97.0)	0.599
Separated/divorced	20 (0.9)	0 (0.0)	0 (0.0)	
Single	44 (1.9)	3 (2.0)	3 (3.0)	
Who lives in at home (lives with), n (%)	*n* = 2334			
Partner	2122 (90.9)	128 (86.5)	79 (79.0)^*^	
Partner and others (e.g. grandparents, friends)	133 (5.7)	9 (6.1)	8 (8.0)	0.000
Others (e.g. grandparents, friends)	50 (2.1)	6 (4.1)	7 (7.0)^*^	
Alone	29 (1.2)	5 (3.4)	6 (6.0)^*^	
Lives with father of the baby, n (%)	*n* = 2342	*n* = 147		
Yes	2224 (95.0)	135 (91.8)	86 (86.0)^*^	0.000
No	118 (5.0)	12 (8.2)	14 (14.0)^*^	
Lives with other children, n (%)	*n* = 2346			
Yes	1113 (47.4)	63 (42.6)	45 (45.0)	0.471
No	1233 (52.6)	85 (57.4)	55 (55.0)	
Region origin, n (%)				
Sub Saharan Africa	393 (16.7)	24 (16.2)	7 (7.0)^*^	
Middle-East/North Africa	988 (42.1)	74 (50.0)	61 (61.0)^*^	0.001
South America	267 (11.4)	13 (8.8)	6 (6.0)	
East Asia/South East Asia	320 (13.6)	26 (17.6)	16 (16.0)	
South Asia	201 (8.6)	6 (4.1)	5 (5.0)	
Europe	178 (7.6)	5 (3.4)	5 (5.0)	
Length of time in Canada (years), n (%)				
< 2 years	603 (25.7)	148 (100.0)^†^	21 (21.0)	0.000
2–5 years	1339 (57.1)	0 (0.0)^†^	65 (65.0)	
> 5 years	405 (17.3)	0 (0.0)^†^	14 (14.0)	
Length of time in Canada, mean months (SD)	40.5 (22.6)	4.1 (2.1)^†^	39.4 (20.4)	0.000
Migration status, n (%)				
Economic immigrant / temporary resident	1046 (44.6)	91 (61.5)^†^	45 (45.0)	
Family sponsored	1203 (51.3)	44 (29.7)^†^	53 (53.0)	0.000
Refugee history / asylum seekers / no status	98 (4.2)	13 (8.8)^†^	2 (2.0)	
Language ability (French and English), n (%)				
Fluent in both/ fluent in one and well in other	667 (28.4)	48 (32.4)	25 (25.0)	
Fluent in one and difficulty or not at all in other	947 (40.3)	46 (31.1)^‡^	47 (47.0)	0.058
Well in one and difficulty or not at all in other	507 (21.6)	30 (20.3)	18 (18.0)	
Difficulty or not at all in both / difficulty in one and not at all in other	226 (9.6)	24 (16.2)^*^	10 (10.0)	

^*^ Denotes significant difference from ‘No transnational care’.

^†^ Denotes significant difference from ‘No transnational care’ and ‘Transnational care, arrived pre-pregnancy’.

^‡^ Denotes significant difference from ‘Transnational care, arrived pre-pregnancy’.

The TPC-arrived pre-pregnancy group also had significant differences compared to the No-TPC group regarding age, region of origin and living arrangements; there was a greater proportion of women ≤ 25 years old (18% vs. 10%) and from the Middle East/North Africa (61% vs. 42%), and a smaller fraction of women from Sub-Saharan Africa (7% vs. 17%); top source countries were Tunisia, Algeria, Morocco, Lebanon, Egypt and Iran. A much larger share of women in the TPC-arrived pre-pregnancy group were not living with the father of the baby (14% vs. 5%) and were living alone (6% vs. 1%) or in other arrangements (e.g., friends) (7% vs. 2%).

The pregnancy and health characteristics by group are reported in Table 2. The three groups did not differ in terms of parity, smoking, drug or alcohol use during pregnancy, maternal general health (infectious disease, anemia) and pregnancy complications. ‘Had previously given birth in Canada’ was similar between the No-TPC (34%) and the TPC-arrived pre-pregnancy (29%) groups; whereas none of the women in the TPC-arrived during pregnancy group had given birth in Canada before. A greater percentage of women in the No-TPC group were overweight pre-pregnancy (25%), had gained excess weight during pregnancy (46%) and were diagnosed with gestational diabetes mellitus (12%) compared to women in the TPC-arrived during pregnancy group (15%, 29% and 5% respectively). Women in the TPC-arrived pre-pregnancy group also had a larger proportion of women who had a ‘normal’ body mass index pre-pregnancy (75%) compared to women in the No-TPC group (61%).

**Table 2:** Pregnancy and health characteristics, N=2595

Characteristic	No transnational care *n* = 2347	Transnational care, arrived during pregnancy *n* = 148	Transnational care, arrived pre-pregnancy *n* = 100	*P* value
Parity, n (%)				
Primiparous	1021 (43.5)	73 (49.3)	50 (50.0)	0.184
Multiparous	1326 (56.5)	75 (50.7)	50 (50.0)	
Gave birth in Canada before, n (%)	*n* = 2336	*n* = 147		
Yes	797 (34.1)	0 (0.0)^*^	29 (29.0)	0.000
No	1539 (65.9)	147 (100.0)^*^	71 (71.0)	
Excess pregnancy weight gain according to the Society of Obstetrician and Gynaecologists of Canada (SOGC) guidelines^†^, n (%)	*n* = 2263	*n* = 137	*n* = 98	0.001
Yes	1038 (45.9)	40 (29.2)^‡^	43 (43.9)	
No	1225 (54.1)	97 (70.8)^‡^	55 (56.1)	
Pre-pregnancy body mass index (BMI), n (%)	*n* = 2287	*n* = 140	*n* = 98	
Underweight	134 (5.9)	11 (7.9)	6 (6.1)	0.010
Normal	1383 (60.5)	96 (68.6)	73 (74.5)^‡^	
Overweight	580 (25.4)	21 (15.0)^‡^	16 (16.3)	
Obese	190 (8.3)	12 (8.6)	3 (3.1)	
Ever smoked during pregnancy, n (%)				
Yes	47 (2.0)	5 (3.4)	3 (3.0)	0.436
No	2300 (98.0)	143 (96.6)	97 (97.0)	
Drug or alcohol use during pregnancy, n (%)				
Yes	26 (1.1)	4 (2.7)	2 (2.0)	0.182
No	2321 (98.9)	144 (97.3)	98 (98.0)	
Poor maternal general health recorded/reported (infectious disease, anemia, chronic illness^§^), n (%)				
Yes (reported/recorded)	796 (33.9)	41 (27.7)	37 (37.0)	0.232
No/info not available	1551 (66.1)	107 (72.3)	63 (63.0)	
Anemia (chronic and/or during pregnancy), n (%)				
Yes (reported/recorded)	465 (19.8)	22 (14.9)	26 (26.0)	0.096
Not reported/ recorded	1882 (80.2)	126 (85.1)	74 (74.0)	
Took multivitamin during pregnancy, n (%)	*n* = 2326		*n* = 99	
Yes	2147 (92.3)	139 (93.9)	89 (89.9)	0.509
No	179 (7.7)	9 (6.1)	10 (10.1)	
Had pregnancy complications^¶^, n (%)				
Yes (reported/recorded)	846 (36.0)	42 (28.4)	29 (29.0)	0.067
Not reported/recorded	1501 (64.0)	106 (71.6)	71 (71.0)	
Gestational diabetes mellitus, n (%)				
Yes (reported/recorded)	292 (12.4)	7 (4.7)^‡^	11 (11.0)	0.019
Not reported/recorded	2055 (87.6)	141 (95.3)^‡^	89 (89.0)	
Prenatal visits ≥ 4 visits, n (%)	*n* = 2300	*n* = 146	*n* = 96	
Yes	2275 (98.9)	133 (91.1)^*^	95 (99.0)	0.000
No	25 (1.1)	13 (8.9)^*^	1 (1.0)	
Prenatal care timely based on arrival in Canada^#^, n (%)	*n* = 2332			
Yes	2073 (88.9)	133 (89.9)	83 (83.0)	0.171
No	259 (11.1)	15 (10.1)	17 (17.0)	
Health insurance, n (%)				0.000
Medicare (public healthcare coverage)	2250 (95.9)	120 (81.1)^*^	93 (93.0)	
Interim Federal Health Program^**^	31 (1.3)	8 (5.4)^‡^	0 (0.0)	
Private only	54 (2.3)	11 (7.4)^‡^	5 (5.0)	
None	12 (0.5)	9 (6.1)^‡^	2 (2.0)	
Reported paying for medical services during pregnancy, n (%)	*n* = 2340	*n* = 147	*n* = 99	0.625
Yes	811 (34.7)	51 (34.7)	39 (39.4)	
No	1529 (65.3)	96 (65.3)	60 (60.6)	

^*^ Denotes significant difference from ‘No transnational care’ and ‘Transnational care, arrived pre-pregnancy’.

^†^https://www.pregnancyinfo.ca/your-pregnancy/healthy-pregnancy/weight-gain-during-pregnancy/.

^‡^ Denotes significant difference from ‘No transnational care’.

^§^ Cardiovascular or respiratory disease, mental illness and/or diabetes.

^¶^ Placental conditions, hypertension, pre-eclampsia, preterm rupture of membranes, intrauterine growth restriction, congenital anomaly, chorioamnionitis, oligohydramnios, hyperemesis gravidarum, urinary tract infections and/or maternal fever.

^#^ As per the Society of Obstetricians and Gynaecologists of Canada guidelines for prenatal care and based on when women arrived in Canada (i.e., first trimester for women in Canada pre-pregnancy, within one month for women who arrived during pregnancy and were < 37 weeks pregnant and within 2 weeks if they were ≥ 37 weeks pregnant.

^**^ Healthcare coverage for asylum-seekers and refugees.

**Fig. 3:**
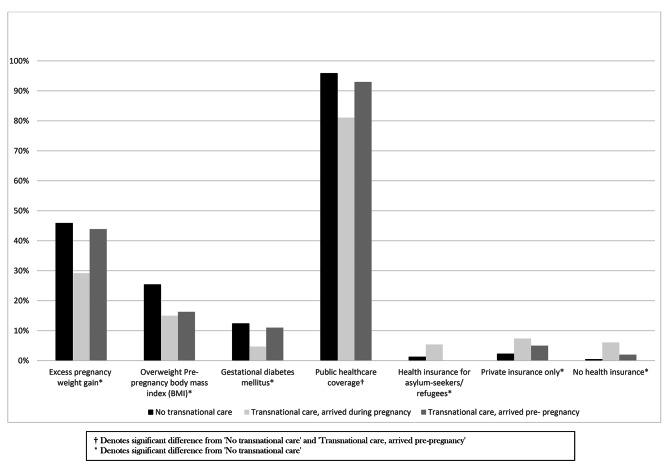
Health characteristics, Transnational care, arrived during pregnancy women vs. No transnational care and Transnational care, arrived pre- pregnancy women

Regarding prenatal visits, 9% of women in the TPC-arrived during pregnancy group had fewer than four prenatal visits in Canada, compared to 1% in the other groups (Table 2). Timely access to prenatal care, however, did not differ between the groups and commencing care late ranged from 10 to 17% across the three groups. Health insurance coverage looked differently for women in the TPC-arrived during pregnancy, with a significantly smaller proportion of women in this group who had provincial Medicare (81% vs. 93–96% in the other groups). A summary of health characteristic differences between TPC-arrived during pregnancy women and No-TPC and TPC-arrived pre-pregnancy women is presented in Fig. 3.

Perceptions and experiences of pregnancy care in Canada by group are described in Table 3. Regardless of group, nearly 90% or more of women always felt healthcare professionals were welcoming, respectful and helpful, felt comfortable asking questions about things they did not understand, and said that decisions were never made without consideration of their wishes. However, women also reported a number of negative experiences and perceptions. Thirty to 44% of women would have liked to use care or services (e.g., prenatal classes, medical tests, social services), and 22–34% of women reported experiencing one or more barriers that prevented them from accessing these services/care (top barriers named were ‘not realizing services were offered’ and ‘having no time’). Ninety-one to 97% said that healthcare professionals did not ask about preferences regarding care; 93–96% were not always asked about their preference for a female or male care-provider; 48–61% had experienced long wait times; and 28–35% felt that healthcare providers could do differently or better.

**Table 3:** Perceptions and experiences of pregnancy care, N=2595

Characteristic	No transnational care *n* = 2347	Transnational care, arrived during pregnancy *n* = 148^*^	Transnational care, arrived pre-pregnancy *n* = 100	*P* value
Would have liked to use services, n (%)	*n* = 2338	*n* = 147		
Yes	704 (30.1)	65 (44.2)^†^	41 (41.0)	0.000
No	1634 (69.9)	82 (55.8)^†^	59 (59.0)	
Did not receive care due to barriers, n (%)	*n* = 2331	*n* = 144	*n* = 99	0.006
No barriers	1823 (78.2)	95 (66.0)^†^	70 (70.7)	
1 barrier	389 (16.7)	36 (25.0)^†^	22 (22.2)	
> 1 barrier	119 (5.1)	13 (9.0)	7 (7.1)	
Enough information was provided, n (%)	*n* = 2345	*n* = 146		
Enough information on all topics	823 (35.1)	43 (29.5)	35 (35.0)	0.215
Not enough information on 1–3 topics	958 (40.9)	57 (39.0)	36 (36.0)	
Not enough information on > 3 topics	564 (24.1)	46 (31.5)	29 (29.0)	
Felt welcomed, n (%)	*n* = 2341	*n* = 145	*n* = 99	
Always	2191 (93.6)	129 (89.0)	89 (89.9)	0.041
Sometimes/rarely/never	150 (6.4)	16 (11.0)	10 (10.1)	
Healthcare professionals were respectful, n (%)	*n* = 2341	*n* = 145	*n* = 99	
Always	2289 (97.8)	137 (94.5)^†^	96 (97.0)	0.041
Sometimes/rarely/never	52 (2.2)	8 (5.5)^†^	3 (3.0)	
Healthcare professionals were helpful, n (%)	*n* = 2339	*n* = 145	*n* = 99	
Always	2210 (94.5)	130 (89.7)^†^	90 (90.9)	0.023
Sometimes/rarely/never	129 (5.5)	15 (10.3)^†^	9 (9.1)	
Was happy with the healthcare received, n (%)	*n* = 2344	*n* = 145	*n* = 99	
Always	2110 (90.0)	124 (85.5)	81 (81.8)^†^	0.010
Sometimes/rarely/never	234 (10.0)	21 (14.5)	18 (18.2)^†^	
The healthcare professionals asked about preferences regarding having a female or male healthcare provider, n (%)	*n* = 2341	*n* = 147		
Always	92 (3.9)	7 (4.8)	7 (7.0)	0.290
Sometimes/rarely/never	2249 (96.1)	140 (95.2)	93 (93.0)	
Understood the information provided by the healthcare professionals, n (%)	*n* = 2344	*n* = 146	*n* = 99	
Always	2100 (89.6)	125 (85.6)	81 (81.8)^†^	0.020
Sometimes/rarely/never	244 (10.4)	21 (14.4)	18 (18.2)^†^	
There was someone who spoke your language or who could interpret for you^‡^, n (%)	*n* = 664	*n* = 51	*n* = 30	
Always	372 (56.0)	31 (60.8)	19 (63.3)	0.605
Sometimes/rarely/never	292 (44.0)	20 (39.2)	11 (36.7)	
Felt worries were taken seriously, n (%)	*n* = 2336	*n* = 144	*n* = 99	
Always	2088 (89.4)	127 (88.2)	83 (83.8)	0.208
Sometimes/rarely/never	248 (10.6)	17 (11.8)	16 (16.2)	
Had to wait too long to receive care, n (%)	*n* = 2345	*n* = 143	*n* = 99	
Never	1204 (51.3)	74 (51.7)	39 (39.4)	0.065
Rarely/sometimes/always	1141 (48.7)	69 (48.3)	60 (60.6)	
Healthcare professionals kept me informed, n (%)	*n* = 2343	*n* = 144	*n* = 99	
Always	2110 (90.1)	129 (89.6)	87 (87.9)	0.771
Sometimes/rarely/never	233 (9.9)	15 (10.4)	12 (12.1)	
Felt comfortable asking about things I did not understand, n (%)	*n* = 2345	*n* = 144	*n* = 99	
Always	2160 (92.1)	134 (93.1)	88 (88.9)	0.458
Sometimes/rarely/never	185 (7.9)	10 (6.9)	11 (11.1)	
Decisions were made by the healthcare professionals without my wishes being taken into account, n (%)	*n* = 2340	*n* = 144	*n* = 99	
Never	2226 (95.1)	138 (95.8)	91 (91.9)	0.320
Rarely/sometimes/always	114 (4.9)	6 (4.2)	8 (8.1)	
Healthcare professionals were encouraging and reassuring, n (%)	*n* = 2342	*n* = 144	*n* = 99	
Always	2069 (88.3)	126 (87.5)	72 (72.7)§	0.000
Sometimes/rarely/never	273 (11.7)	18 (12.5)	27 (27.3)†	
Healthcare professionals spent enough time providing explanations, n (%)	*n* = 2343	*n* = 144	*n* = 99	
Always	1944 (83.0)	121 (84.0)	74 (74.7)	0.097
Sometimes/rarely/never	399 (17.0)	23 (16.0)	25 (25.3)	
Information was provided in your language, n (%)	*n* = 2345	*n* = 146		
Yes	1016 (43.3)	72 (49.3)	34 (34.0)	0.059
No	1329 (56.7)	74 (50.7)	66 (66.0)	
Healthcare professionals asked about plans for baby feeding, n (%)	*n* = 2334	*n* = 145	*n* = 97	
Yes	2012 (86.2)	123 (84.8)	80 (82.5)	0.536
No	322 (13.8)	22 (15.2)	17 (17.5)	
Healthcare professionals asked about preferences about care, n (%)	*n* = 2345	*n* = 145	*n* = 98	
Yes	217 (9.3)	4 (2.8)^†^	6 (6.1)	0.017
No	2128 (90.7)	141 (97.2)^†^	92 (93.9)	
Healthcare professionals offered an interpreting service‡, n (%)	*n* = 749	*n* = 60	*n* = 34	
Yes	91 (12.1)	10 (16.7)	2 (5.9)	0.304
No	658 (87.9)	50 (83.3)	32 (94.1)	
Healthcare professionals could do differently or better, n (%)	*n* = 2316	*n* = 145	*n* = 96	
No	1668 (72.0)	100 (69.0)	62 (64.6)	0.221
Yes	648 (28.0)	45 (31.0)	34 (35.4)	

^*^ Two women were not included in the majority of items because they arrived very late in pregnancy and did not receive any prenatal care in Canada.

^†^ Denotes significant difference from ‘No transnational care’.

^‡^ Only includes women who had English and French language difficulties/no fluency.

^§^ Denotes significant difference from ‘No transnational care’ and ‘Transnational care, arrived during pregnancy’.

Communication was also an issue. Ten to 18% of women did not always understand the information provided by healthcare professionals; 16–25% felt healthcare professionals did not always spend enough time providing explanations; 65–71% reported that they did not receive sufficient information on one or more topics; and 51–66% said that information was not provided in their language. For those with difficulties/no ability in English and French, 90–97% said that they were not offered an interpreting service.

Women in the TPC-arrived during pregnancy group had proportionally more negative experiences and perceptions of pregnancy care compared to women in the No-TPC group. Forty-four percent in the former said that there were care and services that they would have liked to access (compared to 30% in the latter group), and 34% reported not receiving care or services due to one or more barriers (compared to 22%). A slightly larger percentage of women in the TPC-arrived during pregnancy group also felt that the healthcare professionals were not always respectful (6% vs. 2%) and helpful (10% vs. 6%), and almost all women in this group (97%) reported not being asked about preferences regarding their care (compared to 91%).

Women in the TPC-arrived pre-pregnancy group also reported negative perceptions more frequently compared to the No-TPC group. Eighteen percent of women were not always happy with the care they received (compared to 10%), and 18% did not always understand the information provided by healthcare professionals (compared to 10%). Twenty-seven percent (compared to 12%) also did not always feel that the healthcare professionals were encouraging and reassuring.

Lastly, two logistic regression multivariable models were constructed to identify predictors of TPC-arrived pre-pregnancy (see Additional File 3); the models differed slightly depending on whether the 18 women who had said they arrived during pregnancy, but whose length of time in Canada was > 10 months, were included. When these women were included, the length of time in Canada variable was removed from the model and maternal age was significant, whereas when they were excluded, the length of time in Canada variable remained in the model and was significant, and maternal age was non-significant. We therefore decided to retain both variables in the final model. The unadjusted and adjusted ORs for the final model are reported in Additional File 4; the final model is significant (*x*^2^(10) = 55.348, *p* < 0.0010) and shows adequate fit according to the non-significant Hosmer and Lemeshow test (*x*^2^(8) = 6.099, *p* < 0.636). The ROC curve is also reported in Additional File 4; the graph indicates the model has an acceptable discrimination ability (area under the curve = 0.7, 95%CI 0.6, 0.8). The final model results (see Table 4) show that the odds of TPC-arrived pre-pregnancy (vs. No-TPC) were 4.8 times higher for women ‘Not living with the father of the baby’, and 1.2 times higher for women who ‘had more negative perceptions of pregnancy care in Canada (general experiences)’. The odds of TPC-arrived pre-pregnancy also increased with younger maternal age; odds increased 1.1 times (1/0.945) for each year younger. Length of time, 2–5 years, and > 5 years, in Canada (compared to women in Canada < 2 years) are not statistically significant. Region of origin (all regions vs. Europe) contributes to the model, but is also non-significant.

**Table 4:** Final multivariable logistic regression model, Predictors of ‘transnational prenatal care, arrived pre-pregnancy’ vs. No transnational prenatal care, N=2440

Variables	*b*(SE)	Wald	AOR	95%CI
Maternal age	-0.06	5.82^*^	0.95	0.90, 0.99
Length of time in Canada 2–5 years ^a^	0.51	3.61	1.66	0.98, 2.79
Length of time in Canada > 5 years ^a^	0.33	0.81	1.39	0.68, 2.87
Not living with the father of the baby	1.58	19.63^***^	4.85	2.41, 9.75
Region of origin, Sub-Saharan Africa ^b^	-0.90	2.13	0.41	0.12, 1.36
Region of origin, Middle-East/ North Africa ^b^	0.75	2.49	2.13	0.83, 5.42
Region of origin, South America ^b^	-0.30	0.23	0.74	0.22, 2.50
Region of origin, East Asia/South-East Asia ^b^	0.39	0.54	1.48	0.52, 4.18
Region of origin, South Asia ^b^	-0.12	0.03	0.89	0.25, 3.17
Had negative perceptions of pregnancy care in Canada (general experiences)	0.16	13.64^***^	1.18	1.08, 1.28

Note. No transnational prenatal care, n = 2341; Transnational prenatal care, n = 99.

AOR = adjusted odds ratio; 95%CI *=* 95% confidence interval.

^a^. Relative to length of time in Canada less than 2 years.

^b^. Relative to Region of origin = Europe.

^*^p < 0.05 ^***^p < 0.001.

## Discussion

Ten percent of recently-arrived migrant women from LMICs in Montreal received TPC from a healthcare professional, 6% had arrived during pregnancy and 4% were in Canada pre-pregnancy. No previous research examining the rates of TPC use among migrant women were identified, our study is the first, to our knowledge, that reports these prevalence rates. While there are no comparable estimates for our results on TPC associated with migration during pregnancy, our prevalence of TPC-arrived pre-pregnancy can be situated within the larger body of research that has examined transnational healthcare use (i.e., seeking healthcare abroad) among migrants. These studies show rates of transnational healthcare use varying between 9% and 27%, depending on the type of healthcare provider consulted, and the location and migrant population under study [17, 19, 21, 39, 40]. In a representative, population-based study of Polish migrants 15 years or older, in the Netherlands, 24% reported visiting a doctor in Poland within the last year [40]. Similarly, a Danish study found that 27% of adult (aged 18–66 years old) Turkish immigrants used healthcare in a foreign country within the previous year, although when broken down by type of healthcare provider, rates were considerably lower; 15% reported seeing a general practitioner, 12% consulted a specialist, and 9% visited a dentist [21]. A survey of adult Russian immigrants living in Finland, found that 15% of respondents had returned to Russia within the past 12 months to seek care or treatment from a physician [19]and an American study, with Hispanic adults showed 9% of participants had returned to Mexico or another Latin American country during the last 12 months for medical care, dental care, or to purchase medicines or for treatment of an illness or injury [17]. One Canadian study found that 13% of immigrants received dental care outside the country over a four year period [39]. The rate of 4% of TPC use observed in our study may be due to women feeling less mobile during pregnancy compared to seeking care for other health issues. Canada is also geographically distant from many of the home countries of migrants, making return trips costly and time-consuming, and thus travelling may have been unfeasible for most women in our study. Furthermore, migration source countries for Canada differ compared to other migrant-receiving countries, particularly in the province of Quebec, which favors French-speaking migrants, and our sample only included women from LMICs, so cultural and social characteristics of our population may also explain the TPC use pattern observed.

The TPC-arrived during pregnancy women generally were disadvantaged compared to the No-TPC and TPC-arrived pre-pregnancy women. This included a higher proportion of women who had a very low income, a humanitarian/precarious migration status, difficulties/no ability in French and English, and no provincial healthcare coverage. They also more frequently reported facing barriers in accessing care during pregnancy compared to the No-TPC women. These results are consistent with previous research that shows migrant women who are pregnant upon arrival tend be in a more disadvantaged position compared to other childbearing migrant women. For instance, a Canadian cohort study with over 1000 recently-arrived (≤ 5 years) migrant women who gave birth in Montreal and Toronto, showed that over 80% of women who were pregnant upon arrival were asylum-seekers or refugees (73% and 9% respectively) [41]. In the same study, a higher proportion of asylum-seeking and refugee women, compared to immigrant women, also reported having difficulties or no language abilities in both English and French, very low incomes, and no provincial health insurance. Multiple studies have also shown that asylum-seeking and refugee women face numerous barriers in accessing services and care during pregnancy, including a lack of knowledge on existing services and having to deal with competing priorities (e.g., regulating their status, childcare, employment, finding housing) that prevents them from using healthcare [42]. In general, recently-arrived migrants usually prioritize resettling issues, and are less familiar with the healthcare system.

We expect that migrant women with a more challenging context and who arrive during pregnancy would be unlikely to have received prenatal care pre- or during migration. However, we have no information on the quality or extent of care that the TPC-arrived during pregnancy women in our study received before their arrival in Canada, and so it may in fact, have been quite limited. Some women may also have received care while waiting for resettlement in a refugee camp and/or if privately sponsored, they may have received some financial aid to support their access to care before their arrival in Canada. Alternatively, women in this group may have been socially and economically advantaged in their home country and thus they may have had the means to access healthcare pre-migration, or while in transit; given the distance and limited ways to get to Canada from asylum-source regions, only those who have resources tend to be able to make the journey. Although not statistically significant, there was also a higher proportion of women with post-secondary/graduate diplomas in this group. Furthermore, although the TPC-arrived during pregnancy women generally had more at-risk profiles, there was also a greater proportion of women who had an economic/temporary migration status compared to the No-TPC women. The immigration process to Canada is costly and requires to show proof of funds and there is a selection process based on certain criteria, including education level. Therefore these women also likely had the economic and/or social conditions pre-migration that allowed them to easily access prenatal care before coming to Canada. This notion of having a social/economic advantage is further supported by the finding that showed that very few women in the No-TPC group (n = 60) arrived during pregnancy (representing 2% of the total sample), suggesting that women who arrive pregnant in Canada are more likely than not to have received prenatal care before their arrival.

Despite their more at-risk profiles, the TPC-arrived during pregnancy women were healthier in terms of pre-pregnancy BMI, pregnancy weight gain and gestational diabetes, compared to women who only received care in Canada. These results suggest a healthy immigrant effect, where healthier and more socially and economically advantaged migrant women self-selected to migrate [43]. This may be the case since these women did have prenatal care pre-/during migration, and as mentioned above, they do seem to represent somewhat of a select group in terms of their education level. The health advantage may also be due to age, while not statistically significant, this group did have a lower proportion of women aged 35 years and older, compared to the No-TPC women. Moreover, the No-TPC women, who were also recently-arrived migrants (< 8 years in Canada), had rates of weight gain (46%) and gestational diabetes (12%) that are comparable (44%) and higher (3%), respectively when compared to what has been reported for Canadian-born [44–46]. This is consistent with previous research in Canada showing *no* healthy immigrant effect for weight gain and diabetes during pregnancy [45, 47], and thus further suggests that the TPC-arrived during pregnancy women, were indeed a select group.

For TPC-arrived pre-pregnancy women, they were more likely to be younger and were less likely to live with the father of the baby, compared to women who had no TPC. Migrants younger in age may be more mobile because they are generally healthier; this may have been the case in our study since a greater proportion of the TPC-arrived pre-pregnancy women did have a normal BMI pre-pregnancy compared to the No-TPC women. Younger women may also have greater inclinations to make return visits and use healthcare in their home countries because they are less integrated into the destination country and have maintained strong attachments to their home country [18–20, 23, 40, 48]. This hypothesis is supported by the fact that women who used TPC were likely returning to their home countries to be with the father of the baby. Although we do not know for certain where the fathers were, the vast majority of women were married, and thus we can assume that the reason they were not living with the fathers, is because the fathers were in the home countries. Moreover, pregnancy is a special time when women expect and appreciate greater levels of support from family. Women in our study therefore may also have travelled to spend more time with their extended family and to rely on their networks back home, and while there, accessed healthcare [25].

Women who were residing in Montreal pre-pregnancy and who sought transnational care were also more likely to report negative care experiences compared to those who received no TPC. Experiences of discrimination within healthcare encounters [19] and poorer perceived quality of healthcare [17] in the destination country have previously been shown to be associated with transnational healthcare use. Facing barriers to care in the destination country, including long wait times, and having established relationships with healthcare providers in the home country, have also been reported by migrants as reasons for seeking transnational healthcare [20, 23]. Women therefore may have accessed care in the home country in response to local barriers and/or because they had unmet cultural needs and expectations of healthcare professionals and preferred their care-providers in their home countries. However, if the use of care abroad was more a matter of timing or convenience because women’s primary reason for returning to the home country was to visit family, negative perceptions may simply have been the result of comparing their care experiences across healthcare systems.

Overall, regardless of women’s TPC use, perceptions of pregnancy care in Canada were generally positive, although an important share of women started prenatal care late, experienced barriers in accessing care and services during pregnancy, had communication issues, felt they waited too long for care, and felt that care-providers did not inquire about their preferences, including their desire to have a female care-provider. A study that examined Chinese migrant women’s experiences with maternity services in Toronto, Canada also highlighted that most women were satisfied with the care received; women mentioned the caring attitude of nurses, and greater levels of privacy and cleanliness of facility compared to what was expected back in their origin country [49]. Similarly, in a Canadian study in British Columbia, newcomer, Punjabi mothers generally described positive interactions with healthcare providers [14]. Alternatively, other research suggests that some migrants may not be comfortable sharing negative views on healthcare providers, or identifying shortcomings in the receiving-country healthcare system, which could explain the generally positive accounts from women in our study, especially since they were still in the hospital when asked about their perceptions and experiences [50].

Regarding the challenges reported by the migrant women in our study, these are consistent with what has been found in previous research. Long wait times, not being asked about preferences, difficulties in accessing care, language barriers, including information not being provided in one’s language, and/or not being offered an interpreter, and other communication related challenges, such as information not being sufficient or care-providers not spending adequate time to explain, are commonly known issues experienced by migrant women during maternity care [7, 51–53]. However, some of these issues are not unique to migrants; receiving-country women have also expressed problems with wait times and care interactions that are rushed, and have conveyed that they would like more time, information and personalized care [7]. Lastly, a systematic review on migrant women’s utilization of prenatal care showed that late initiation rates are more common for migrant women when compared to native-born women [4]. According to the Canadian maternity experiences survey conducted in 2006, 6.2% of non-recent immigrant women (> 5years in Canada), 6.9% of recent immigrant women (≤ 5 years in Canada) and 4.7% of Canadian-born women, initiated prenatal care after the first trimester [54]. These rates are lower than what was observed in our study (i.e., 10–17% of women did not receive timely prenatal care), suggesting that the migrant women in our research may have faced additional barriers to prenatal care, or in the case for women who sought care abroad, delays may have been due to their TPC use.

### Implications for care

The use of prenatal care in another country, whether associated with migration during pregnancy, or due to women actively seeking care abroad, may result in fragmented care or contradictions in diagnoses, treatments, prescriptions and care recommendations between the different care-providers and thus may have negative health consequences. Alternatively, seeking care in the home country may address inadequacies regarding support and cultural preferences in prenatal care delivered in the receiving-country. To reduce the potential for harm and to ensure a continuity of care, healthcare providers should therefore inquire about care, support and other services received outside of the country during pregnancy.

The results of the study also point to a need to address barriers to care, especially for women who arrive during pregnancy, including early receipt of information on services and prenatal care available, and details on where and how to access these. Healthcare providers could also do more to address language and communication issues, including offering interpreters and translated materials, and taking more time to deliver and explain information. To better respond to migrant women’s expectations and needs, healthcare providers should also ask about preferences [55]. At the system level, long wait times need to be resolved.

### Limitations and strengths

There are several limitations to this study, and these should be kept in mind when interpreting the results. We only had a crude measure of TPC. We do not know exactly how women interpreted the questions on prenatal care and locations of care since there were no questions explicitly asking details about the prenatal care. We assumed that most women only considered in-person care in another country when responding to these questions. However, it is possible that some women also deemed virtual interactions with a healthcare professional or medicines sent from a healthcare provider, as having received prenatal care in another country. We also did not have any information on the content of the care including which healthcare professionals were accessed, and when and to what extent (number of contacts) care in the other country was received.

Secondly, there may have been some error in how women were classified into the TPC groups since recall on time in Canada and pregnancy status might not have been precise. Women might also have misunderstood the question regarding pregnancy status upon arrival and responded based on a previous pregnancy or could have said yes if they had been visiting abroad and returned from a visit, pregnant. We also found a few discrepancies in the length of time in Canada variable, and so this question might also have been improperly recorded in some instances, or depending on women’s migration trajectories (i.e., if women had come and gone between Canada and their home country), they might have answered this question inconsistently. We also categorized women based on the assumption that women who had said they migrated during pregnancy, received TPC as a result of their migration during pregnancy, and that women in Canada pre-pregnancy, had received it because they intentionally sought out care abroad- we cannot confirm whether this is true since we had no information on why women received TPC.

In general, most of the data were susceptible to recall error since they were based on maternal report post-birth, which is a relatively chaotic time for women. Medical records are limited as a data source since information is inconsistently recorded, and often, charting is done by exception. Because our study was a secondary analysis, not all variables were available in an ideal format and we did not have information on transnational social/family ties, which would have been a relevant variable for this study.

Lastly, the sample sizes for the TPC groups were quite small and so type two error might have occurred. The small sample also limited the number of variables that could be included in the multivariable logistic regression. Due to the cross-sectional design, the temporality of the relationships between certain variables and TPC-arrived pre-pregnancy, cannot be confirmed. Overall, the results (prevalence of TPC, the profiles of TPC women and the predictors of TPC-arrived pre-pregnancy) may not be generalizable to other countries, given the different migration patterns observed in Canada, and in the province of Quebec specifically.

This study has a number of strengths. In the MFMC study all women who gave birth during the recruitment period were considered for participation and refusal and withdrawal rates were small (11% and 0.5% respectively). If there were differences in participation rates between women who had TPC versus those who did not, it is likely that their profiles and/or perceptions of care were more extreme (i.e., more at-risk profiles and/or more negative views on care) so any biases in our results would have been towards the null. We used two variables (‘arrived during pregnancy’ and ‘length of time in Canada’) to create our TPC groups. We conducted multivariable models with and without the 18 women who may have been misclassified to determine if this might have had an effect on the results. The MFMCQ was a culturally validated tool and available in eight languages, and there is no reason to suspect that information bias was an issue. This is the first study of which we are aware that quantitatively examines TPC from a healthcare professional among migrant women in a high-income country; it therefore sets the stage for, and will inform the design of, future studies.

In future research, migrant women should be followed prospectively and details gathered on their actual use of transnational maternity care, both prenatally and postnatally, including international travel to use services in person as well as other forms of transnational care and support received (e.g., advice, medical information and medicines sent from abroad). Data should also be collected on women’s motivations and experiences of seeking transnational care. It would also be worthwhile to examine the relationships between transnational maternity care and maternal and infant outcomes. Future studies should also include women from non-LMICs of origin, and women who have been in Canada longer, as they may have different prevalence and patterns of use of transnational maternity care.

## Conclusions

An important proportion (10%) of recently-arrived migrant women from LMICs who are giving birth in Montreal, Canada are mobile during pregnancy and receiving prenatal care in a country other than Canada. Women who migrate during pregnancy and receive TPC (6%) generally represent a more disadvantaged group compared to TPC-arrived pre-pregnancy and No-TPC women, in terms of income level, migration status, French and English language abilities, and healthcare coverage. They also experience more barriers accessing care and services during pregnancy compared to No-TPC women. However, they also have a higher proportion of economic migrants and are healthier, suggesting a healthy immigrant effect, whereby these women self-select to migrate during pregnancy because they have the means and capacity to do so. TPC-arrived pre-pregnancy women (4%), also appear to have a health advantage (compared to No-TPC women) which may facilitate their use of pregnancy care abroad. They may also be motivated to return to the home country for healthcare during pregnancy due to a need for family and social support and/or due to preferences for the care-providers in the home country. Overall, experiences and perceptions of pregnancy care in Canada are positive, although late prenatal care, barriers in accessing care and services during pregnancy, communication issues, long wait times, and care-providers not asking about preferences, are of concern for a significant amount of migrant women. In general, TPC women, regardless of when they arrive in Canada, are more likely to report negative views compared to No-TPC women. Given their particularly disadvantaged status, TPC-arrived during pregnancy women may need additional support and care to overcome access barriers.

## Electronic supplementary material

Below is the link to the electronic supplementary material.

**Additional file 1**

**Additional file 2**

**Additional file 3**

**Additional file 4**

## Data Availability

The data that support the findings of this study are available from the corresponding author (LM), upon reasonable request.
