# Supplementary material for: Transnational prenatal care among migrant women from low-and-middle-income countries who gave birth in Montreal, Canada
Source: BMC Pregnancy Childbirth. 2023 Apr 26;23:292. doi: 10.1186/s12884-023-05582-w (PMC10131434; doi:10.1186/s12884-023-05582-w)
Supplement: Supplementary file 2 — Additional file 2 [file 12884_2023_5582_MOESM2_ESM.docx]

**Additional File 2: Variables included in the model**

| **Variable** | **SPSS Variable name** | **Comments** | **Hypothesis** |
| --- | --- | --- | --- |
| *Parity* | Primip  1=Primip; 2=Multip | Mutip =2 as reference | Primips more likely to transcare |
| *Maternal age* | MatAgeYrs | continuous | Younger more likely to transcare |
| *Length of time in Canada* | LOTcatgs  1= < 2 years  2= 2-5 years;  3= > 5 years | < 2 years =1  as reference | 2-5 years more likely to transcare |
| *Education* | MFMCQ102  1=primary; 2=secondary; 3=postsecondary; 4=grad; | 1=primary; 2=secondary; 5=none **vs.**  3=postsecondary; 4=grad  Less education as reference | More educated more likely to transcare |
| *Living with father of baby* | LivesWbbFather  1= Yes; 2=No | Yes =1 as reference | Not living with father of baby more likely to transcare |
| *Region of origin* | Region  1=Sub-Saharan Africa; 2=Middle-East/N. Africa; 3=South America;  4=E. Asia/S.E. Asia; 5=South Asia;  6=E. Europe | Europe=6 as reference | From the Middle-East/North Africa more likely to transcare;  From Sub-Saharan Africa and South America less likely to transcare |
| *Paying for medical services during pregnancy* | PAIDmedServs  1=Yes; 2=No | No =2 as reference | Paid for services more likely to transcare |
| *Pregnancy complications^*^* | PregCompl  1=Yes (reported/recorded);  2= Not reported/recorded | Yes =1 as reference | No complications more likely to transcare |
| *Perceptions of pregnancy care in Canada (general experiences)*^*^ | Composite variable  (Score out of 14)  (see below for details) | Prorated Score | Higher scores (more negative perceptions) more likely to transcare |
| *Perceptions of pregnancy care in Canada (language/communication related)*^*^ | Composite variable  (Score out of 8)  (see below for details) | Prorated Score | Higher scores (more language/communication issues) more likely to transcare |

^*^could be an outcome of transnational pregnancy care

**Composite variable: Perceptions of pregnancy care (general experiences)**

| Would have liked to use services | WantedServs  **1=Yes**; 2=No |
| --- | --- |
| Did not receive care due to barriers | BarriersExp  1=No barriers; 2=1 barrier; **3= > 1 barrier** |
| Felt welcomed | MFMCQ48a  1=Always; **2=Sometimes; 3=Rarely; 4=Never** |
| Healthcare professionals were respectful | MFMCQ49a  1=Always; **2=Sometimes; 3=Rarely; 4=Never** |
| Healthcare professionals were helpful | MFMCQ50a  1=Always; **2=Sometimes; 3=Rarely; 4=Never** |
| Was happy with the healthcare received | MFMCQ51a  1=Always; **2=Sometimes; 3=Rarely; 4=Never** |
| Healthcare professionals asked about preferences about having a female or male care provider | MFMCQ54a  1=Always; **2=Sometimes; 3=Rarely; 4=Never** |
| Felt worries were taken seriously | MFMCQ69a  1=Always; **2=Sometimes; 3=Rarely; 4=Never** |
| Had to wait too long to receive care | MFMCQ70a  1= Never; **2= Rarely ; 3= Sometimes; 4= Always** |
| Decisions were made by the healthcare professionals without wishes being taken into account | MFMCQ73a  1= Never; **2= Rarely ; 3= Sometimes; 4= Always** |
| Healthcare professionals were very encouraging and reassuring | MFMCQ74a  1=Always; **2=Sometimes; 3=Rarely; 4=Never** |
| Healthcare professionals asked about baby feeding plans | MFMCQ15  1=Yes; **2=No** |
| Healthcare professionals asked about preferences about care | MFMCQ16  1=Yes; **2=No** |
| Healthcare professionals could do differently or better | DiffBettPreg  1=No; **2=Yes** |
| **TOTAL SCORE**  **For each item, score as 0 or 1 (1 if blue) and then add to obtain a total score** | /14 |

**Composite variable: Perceptions of care (language/communication related)**

| Had enough information | EnuffInfo  1=Enough info on all topics asked;  2=Not enough info on one topic;  **3=Not enough on 2 or 3 topics;**  **4=Not enough info on >3 topics** |
| --- | --- |
| Understood the information provided by the healthcare professionals | MFMCQ55a  1=Always; **2=Sometimes; 3=Rarely; 4=Never** |
| There was someone who spoke your language and could interpret for you | MFMCQ58a  0=N/A; 1=Always; **2=Sometimes; 3=Rarely; 4=Never** |
| Healthcare professionals kept you informed | MFMCQ71a  1=Always; **2=Sometimes; 3=Rarely; 4=Never** |
| I felt comfortable asking about things I did not understand | MFMCQ72a  1=Always; **2=Sometimes; 3=Rarely; 4=Never** |
| Healthcare professionals spent enough time providing explanations | MFMCQ75a  1=Always; **2=Sometimes; 3=Rarely; 4=Never** |
| Healthcare professional gave you information in your language | MFMCQ13  1=Yes; **2=No** |
| Healthcare professionals offered an interpreting service | InterpPreg  0= N/A; 1=Yes; **2=No** |
| **TOTAL**  **For each item, score as 0 or 1 (1 if blue) and then add to obtain a total score** | /8 |
