# Supplementary material for: Transnational prenatal care among migrant women from low-and-middle-income countries who gave birth in Montreal, Canada
Source: BMC Pregnancy Childbirth. 2023 Apr 26;23:292. doi: 10.1186/s12884-023-05582-w (PMC10131434; doi:10.1186/s12884-023-05582-w)
Supplement: Supplementary file 3 — Additional file 3 [file 12884_2023_5582_MOESM3_ESM.docx]

**Additional File 3**

**Multivariable logistic regression model: Variables associated with ‘transnational prenatal care, arrived pre-pregnancy’,**

**All participants included; N= 2440 (No transnational prenatal care, n= 2341 and Transnational prenatal care, n=99)**

|  | | | | | | | | | |
| --- | --- | --- | --- | --- | --- | --- | --- | --- | --- |
|  | | B | S.E. | Wald | df | Sig. | Exp(B) | 95% C.I.for EXP(B) | |
|  |  |  |  |  |  |  |  | Lower | Upper |
|  | MatAgeYrs | -,048 | ,023 | 4,567 | 1 | ,033 | ,953 | ,911 | ,996 |
|  | Region origin |  |  | 20,121 | 5 | ,001 |  |  |  |
|  | Sub-Saharan Africa | -,946 | ,617 | 2,352 | 1 | ,125 | ,388 | ,116 | 1,301 |
|  | Middle-East/ North Africa | ,706 | ,477 | 2,194 | 1 | ,139 | 2,026 | ,796 | 5,159 |
|  | South America | -,292 | ,618 | ,224 | 1 | ,636 | ,746 | ,222 | 2,507 |
|  | East Asia / South East Asia | ,356 | ,530 | ,452 | 1 | ,502 | 1,428 | ,505 | 4,036 |
|  | South Asia | -,157 | ,647 | ,059 | 1 | ,808 | ,855 | ,241 | 3,036 |
|  | Not Living with father of the baby | 1,606 | ,354 | 20,546 | 1 | ,000 | 4,985 | 2,489 | 9,985 |
|  | Negative perceptions of general experience during pregnancy- Prorated : Raw score * number of items (total) divided by number of valid answers | ,155 | ,043 | 12,681 | 1 | ,000 | 1,168 | 1,072 | 1,271 |
|  | Constant | -2,672 | ,871 | 9,405 | 1 | ,002 | ,069 |  |  |
| Reference for Region: Europe  B = coefficient for the constant (the intercept)  S.E. = standard error  Wald =Wald chi-square test  Sig = significance, the p-value  Df = degrees of freedom  Exp(B) = exponentiation of the B coefficient, which is an odds ratio  95% C.I. = 95% confidence interval | | | | | | | | | |

**Multivariable logistic regression model: Variables associated with ‘transnational prenatal care, arrived pre-pregnancy’,**

**Excluding the 18 women who said they arrived during pregnancy, but whose length of time in Canada was > 10 months; N= 2423 (No transnational prenatal care, n= 2341 and Transnational prenatal care, n=82)**

|  | | | | | | | | | |
| --- | --- | --- | --- | --- | --- | --- | --- | --- | --- |
|  | | B | S.E. | Wald | df | Sig. | Exp(B) | 95% C.I.for EXP(B) | |
|  |  |  |  |  |  |  |  | Lower | Upper |
|  | MatAgeYrs | -,046 | ,026 | 3,278 | 1 | ,070 | ,955 | ,908 | 1,004 |
|  | Length of time in Canada - categories |  |  | 5,718 | 2 | ,057 |  |  |  |
|  | Length of time in Canada –  2-5 years | ,716 | ,305 | 5,518 | 1 | ,019 | 2,046 | 1,126 | 3,720 |
|  | Length of time in Canada –  > 5 years | ,440 | ,421 | 1,094 | 1 | ,296 | 1,553 | ,681 | 3,544 |
|  | Region origin |  |  | 18,556 | 5 | ,002 |  |  |  |
|  | Sub-Saharan Africa | -1,077 | ,736 | 2,139 | 1 | ,144 | ,341 | ,080 | 1,442 |
|  | Middle-East/ North Africa | ,843 | ,528 | 2,544 | 1 | ,111 | 2,323 | ,825 | 6,542 |
|  | South America | -,089 | ,657 | ,018 | 1 | ,892 | ,915 | ,252 | 3,316 |
|  | East Asia / South East Asia | ,293 | ,600 | ,239 | 1 | ,625 | 1,341 | ,414 | 4,347 |
|  | South Asia | -,091 | ,720 | ,016 | 1 | ,899 | ,913 | ,223 | 3,742 |
|  | Not living with father of the baby | 1,187 | ,448 | 7,026 | 1 | ,008 | 3,278 | 1,363 | 7,887 |
|  | Negative perceptions of general experience during pregnancy- Prorated : Raw score * number of items (total) divided by number of valid answers | ,179 | ,046 | 14,834 | 1 | ,000 | 1,196 | 1,092 | 1,309 |
|  | Constant | -3,585 | ,967 | 13,753 | 1 | ,000 | ,028 |  |  |
| Reference for Region: Europe; Reference for length of time in Canada: < 2 years  B = coefficient for the constant (the intercept)  S.E. = standard error  Wald =Wald chi-square test  Sig = significance, the p-value  Df = degrees of freedom  Exp(B) = exponentiation of the B coefficient, which is an odds ratio  95% C.I. = 95% confidence interval | | | | | | | | | |
