# Supplementary material for: Transnational prenatal care among migrant women from low-and-middle-income countries who gave birth in Montreal, Canada
Source: BMC Pregnancy Childbirth. 2023 Apr 26;23:292. doi: 10.1186/s12884-023-05582-w (PMC10131434; doi:10.1186/s12884-023-05582-w)

**Additional File 4**

**Predictors of ‘transnational prenatal care, arrived pre-pregnancy’ vs. No-Transnational Prenatal Care, Unadjusted and adjusted odds ratios**

| **Variable** | **OR (95%CI)** | **AOR (95%CI)** |
| --- | --- | --- |
| Primiparous  Multiparous | 1.30 (0.87, 1.94)  1.00 |  |
| Maternal age (Years) | 0.95 (0.91, 0.99) | 0.95 (0.90, 0.99) |
| Length of time in Canada  2-5 years  > 5 years  < 2 years | 1.39 (0.84, 2.30)  0.99 (0.50, 1.98)  1.00 | 1.66 (0.98, 2.79)  1.39 (0.68, 2.87)  1.00 |
| Postsecondary education or higher  Primary, secondary, or no education | 0.69 (0.37, 1.31)  1.00 |  |
| Not living with the father of the baby  Living with the father of the baby | 3.07 (1.69, 5.56)  1.00 | 4.85 (2.41, 9.75)  1.00 |
| Region of origin  Sub-Saharan Africa  Middle-East/ North Africa  South America  East Asia/South-East Asia  South Asia  Europe | 0.63 (0.20, 2.03)  2.20 (0.87, 5.55)  0.80 (0.24, 2.66)  1.78 (0.64, 4.94)  0.89 (0.25, 3.11)  1.00 | 0.41 (0.12, 1.36)  2.13 (0.83, 5.42)  0.74 (0.22, 2.50)  1.48 (0.52, 4.18)  0.89 (0.25, 3.17)  1.00 |
| Paid for medical services during pregnancy  Did not pay for medical services during pregnancy | 1.23 (0.81, 1.85)  1.00 |  |
| Had pregnancy complications  Pregnancy complications not recorded or reported | 1.38 (0.89, 2.14)  1.00 |  |
| Negative perceptions of pregnancy care in Canada (general experiences) (Prorated score out of 14 items) | 1.16 (1.07, 1.26) | 1.18 (1.08, 1.28) |
| Negative perceptions of pregnancy care in Canada (language/communication) (Prorated score out of 8 items) | 1.14 (1.01, 1.29) |  |

N= 2440, No transnational prenatal care, n= 2341 and Transnational prenatal care, n=99

Abbreviations: *OR* odds ratio; *AOR* adjusted odds ratio; *95% CI* 95% confidence interval

**The receiver operating characteristic (ROC) curve for the final logistic regression multivariable model results predicting odds of ‘Transnational prenatal care -arrived pre-pregnancy’ vs. No-Transnational prenatal care**


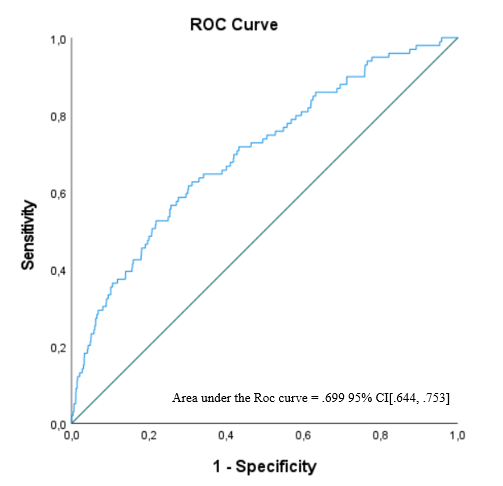

Supplement: Supplementary file 4 — Additional file 4 [file 12884_2023_5582_MOESM4_ESM.docx]
